# Influence of Surface Isolation Layers on High-Voltage Tolerance of Small-Pitch 3D Pixel Sensors

**DOI:** 10.3390/s25144478

**Published:** 2025-07-18

**Authors:** Jixing Ye, Gian-Franco Dalla Betta

**Affiliations:** 1 Dipartimento di Ingegneria Industriale, Università degli Studi di Trento, 38123 Trento, Italy; jixing.ye@unitn.it; 2 Trento Institute for Fundamental Physics and Applications—Istituto Nazionale di Fisica Nucleare (TIFPA-INFN), 38123 Trento, Italy

**Keywords:** 3D pixel sensors, TCAD simulation, breakdown voltage, radiation damage effects

## Abstract

In recent years, 3D pixel sensors have been a topic of increasing interest within the High Energy Physics community. Due to their inherent radiation hardness, demonstrated up to a fluence of $3\times 10^{16}$ 1 MeV equivalent neutrons per square centimeter, 3D pixel sensors have been used to equip the innermost tracking layers of the ATLAS and CMS detector upgrades at the High-Luminosity Large Hadron Collider. Additionally, the next generation of vertex detectors calls for precise measurement of charged particle timing at the pixel level. Owing to their fast response times, 3D sensors present themselves as a viable technology for these challenging applications. Nevertheless, both radiation hardness and fast timing require 3D sensors to be operated with high bias voltages on the order of ∼150 V and beyond. Special attention should therefore be devoted to avoiding problems that could cause premature electrical breakdown, which could limit sensor performance. In this paper, TCAD simulations are used to gain deep insight into the impact of surface isolation layers (i.e., p-stop and p-spray) used by different vendors on the high-voltage tolerance of small-pitch 3D sensors. Results relevant to different geometrical configurations and irradiation scenarios are presented. The advantages and disadvantages of the available technologies are discussed, offering guidance for design optimization. Experimentalmeasurements from existing samples based on both isolation techniques show good agreement with simulated breakdown voltages, thereby validating the simulation approach.

## 1. Introduction

The upgrade of the Large Hadron Collider (LHC) [1] to the High Luminosity phase (HL-LHC) [2] will expose tracking detectors to unprecedented levels of radiation, thereby requiring innovative designs capable of withstanding these harsh conditions. Among possible sensor technologies, 3D sensors [3] are particularly suitable. The high radiation hardness of these devices, which was proved up to very high fluences of the order of $3\times 10^{16}$ 1 MeV equivalent neutrons per square centimeter ($n_{\mathrm{eq}}$ ${\mathrm{cm}}^{-2}$) [4], is due to their unique geometry [5] featuring vertical electrodes penetrating through the silicon substrate. In this fashion, the inter-electrode distance can be decoupled from the substrate thickness, and choosing a value comparable to the effective drift length of charge carriers ensures high signal efficiency even in the presence of severe charge trapping. The first application of 3D pixel sensors in High Energy Physics (HEP) has been in the Insertable B-Layer (IBL) [6] of the ATLAS (A Toroidal LHC ApparatuS) experiment at the LHC [7]. Leveraging this successful experience, a new generation of small pitch 3D sensors has been developed by IMB-CNM (Barcelona, Spain) [8], SINTEF (Oslo, Norway) [9], and FBK (Trento, Italy) [10] to meet the demands of both general purpose experiments at the HL-LHC, namely the ATLAS and the CMS (Compact Muon Solenoid) [11]. These sensors are based on single-sided technologies with a single readout electrode and feature pixel sizes of 25 × 100 μ$m^{2}$ and 50 × 50 μ$m^{2}$, referred to as 25 × 100−1E and 50 × 50−1E, respectively [12]. In beam tests, these sensors have demonstrated a hit efficiency of approximately 97% after fluences up to ∼$10^{16}$ $n_{\mathrm{eq}}$ ${\mathrm{cm}}^{-2}$, confirming their ability to perform effectively in the challenging radiation environment of the HL-LHC. For this reason, 25 × 100−1E and 50 × 50−1E have been chosen to equip the barrel and the end-cap region, respectively, of the innermost pixel layer of the ATLAS Inner Tracker (ITk) [13]. Moreover, 25 × 100−1E pixel sensors will equip the first layer of the central barrel of the CMS Inner Tracker [14].

In addition to extreme radiation hardness, 3D sensors also feature very fast response [15], which makes them appealing for the next generation of vertex detectors, where a precise measurement of charged particle timing (i.e., 4D-tracking) at the pixel level is necessary. Excellent timing resolution values were already reported for 3D sensor prototypes with both columnar electrodes (∼30 ps, [16]) and trench electrodes (∼10 ps, [17]). Optimization of radiation hardness and timing resolution requires full substrate depletion and high drift velocity of charge carriers, thus requiring high sensor bias voltage. Special attention should therefore be devoted to avoiding problems that could cause premature electrical breakdown, which could otherwise jeopardize the sensors’ performance. This is particularly important for small-pitch 3D sensors, where the proximity between the electrodes leads to relatively high electric fields already at low voltage.

Breakdown properties of 3D sensors have been the subject of several studies at the analytical level [18], device simulation level [19,20,21,22,23], and experimental level [24,25]. All studies point to critical regions in the device where high electric field peaks are responsible for electrical breakdown, namely the ${\mathrm{SiO}}_{2}$/Si interfaces and the electrode tips, in cases where the readout electrodes do not pass all the way through the substrate. However, the influence of different surface isolation technologies on the high-voltage tolerance of 3D sensors has not been studied in detail yet, a gap that this paper aims to fill with the aid of TCAD (Technology Computer-Aided Design) simulations. TCAD enables prediction of device performance based on technological and geometrical parameters under varying operating conditions (e.g., bias voltage, temperature, and radiation exposure). By providing deep insight into detector behavior, TCAD helps enhance performance, reliability, and radiation hardness while reducing development time and cost, so it represents the ideal tool for this study.

Short of a few exceptions, 3D sensors are fabricated using p-type substrates with $n^{+}$ readout electrodes in order to exploit the higher drift velocity and the lower trapping probability of electrons with respect to holes. In this configuration, isolation structures are required to interrupt the surface electron layer induced by the positive oxide charge, which would otherwise short the $n^{+}$ electrodes. Two different techniques are normally utilized in 3D sensors: a uniform p-type implantation, known as p-spray [26], and a patterned p-type implantation, known as p-stop [27]. A combination of the two is also possible, as demonstrated by FBK [28], but this adds further complication to the already difficult process.

Each fabrication facility has its own isolation technique preference: IMB-CNM uses p-stop [29], whereas SINTEF [30] and FBK [31] use p-spray. The reasons for these choices have not been disclosed. In fact, FBK had made a direct comparison of p-stop and p-spray techniques in 3D Single-Type-Column Detectors [32], also studying their impact on sensor noise and signal in response to laser pulses [33], but the oversimplified structure and very different electric field distribution of these early prototypes prevent any conclusion for full 3D sensors.

It should be stressed that both p-stop and p-spray are not novel techniques and were originally applied to the n-side of double-sided microstrip sensors. Only later were they adapted for other types of sensors, including 3D sensors. A comprehensive review of the advantages and disadvantages of these isolation techniques based on TCAD simulations of planar strip sensors on p-type substrates can be found in [34], which inspired the work presented in this paper. While some general considerations reported in [34] are also applicable to 3D sensors, the particular geometry of these devices significantly affects the behavior of the isolation structures.

P-stop and p-spray are floating regions; their electric potential depends on the punch-through effect [35] taking place between them and the backside $p^{+}$ region in planar sensors. Conversely, the electric potential of the p-stop (p-spray) region in 3D sensors is completely different because of the short (non-existent) distance from the vertical bias electrodes. This highlights the need for a systematic TCAD simulation study of different isolation techniques for small-pitch 3D pixel sensors, which is the objective of this paper. In addition, the present study demonstrates that bulk damage effects, which were not considered in [34] and are of utmost interest for the applications of 3D sensors, have a strong impact on the high-voltage tolerance of these devices. For the above reasons, this study represents an important contribution to the full understanding of the functioning of these devices as well as to their optimization.

The remaining part of the paper is organized as follows. Section 2 introduces the concept of the devices under study as well as the simulation approach. Section 3 covers the geometrical effects of p-stop on the high-voltage tolerance of the devices, including different p-stop positions and widths, while the results for p-spray isolation are presented in Section 4. Section 5 illustrates how the presence of the junction electrode tip affects the sensors’ overall performance through simulation and reports a comparison between simulations and experimental data in order to validate the simulation approach. The impact of the surface isolation layers on the capacitance is reported in Section 6, followed by conclusions and outlook in Section 7.

## 2. Simulation Setup

### 2.1. Structure of Small-Pitch 3D Pixel Sensors

**Figure 1:**
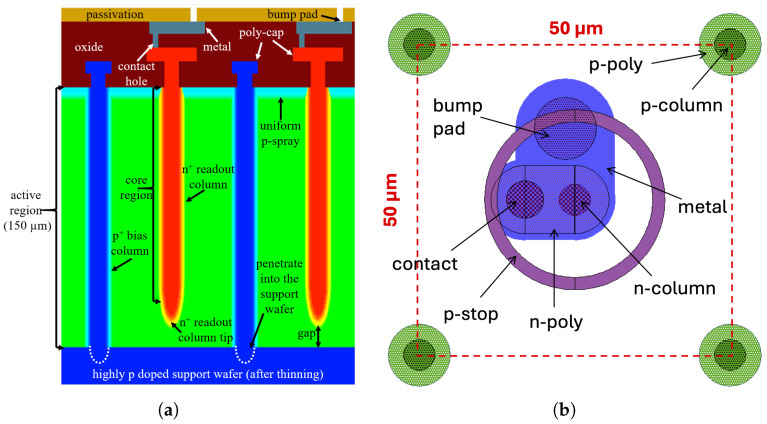
Device under study: (**a**) schematic cross-section with p-spray present at the interface (not to scale); (**b**) possible layout of a 50 × 50−1E pixel with p-stop.

Small-pitch 3D pixel sensors are the state-of-the-art in 3D sensors. They are fabricated on p-type Si-Si Direct Wafer Bonded substrates, as illustrated in Figure 1a. The support wafer is made sufficiently thick to ensure mechanical strength, allowing for the use of thin active layers—down to 150 μm—in the 3D pixel sensors relevant to the HL-LHC upgrade of the ATLAS and the CMS. This feature is essential for downscaling the pixel size that would otherwise be limited by the electrode aspect ratio. All electrodes are fabricated from the frontside using the Deep Reactive Ion Etching (DRIE) method. The bias (ohmic) electrodes are etched deep into the highly doped p-type support wafer to enable direct biasing from the backside, while the readout (junction) electrodes are kept at a safe distance from the backside to prevent electrical breakdown at very low voltage. The vertical electrodes are entirely or partially filled with doped poly-Si, which also forms caps at the column openings, acting as field plates.

Compared to the double-sided 3D sensors of the ATLAS IBL [6], which have a pixel size of 50 × 250 μ$m^{2}$, small-pitch 3D pixel sensors have greatly reduced pixel sizes to meet the occupancy and radiation hardness requirements at the HL-LHC. To study the influence of different isolation techniques on small-pitch 3D pixel sensors, the 50 × 50−1E design is considered in this paper. Figure 1b shows a possible pixel layout with p-stop, where the columnar electrodes and other details such as poly-Si caps on both types of electrodes are visible. Among the available p-stop designs [36], only the individual (atoll) p-stop design is considered due to limited space in the pixel area.

### 2.2. Simulation Approach

**Figure 2:**
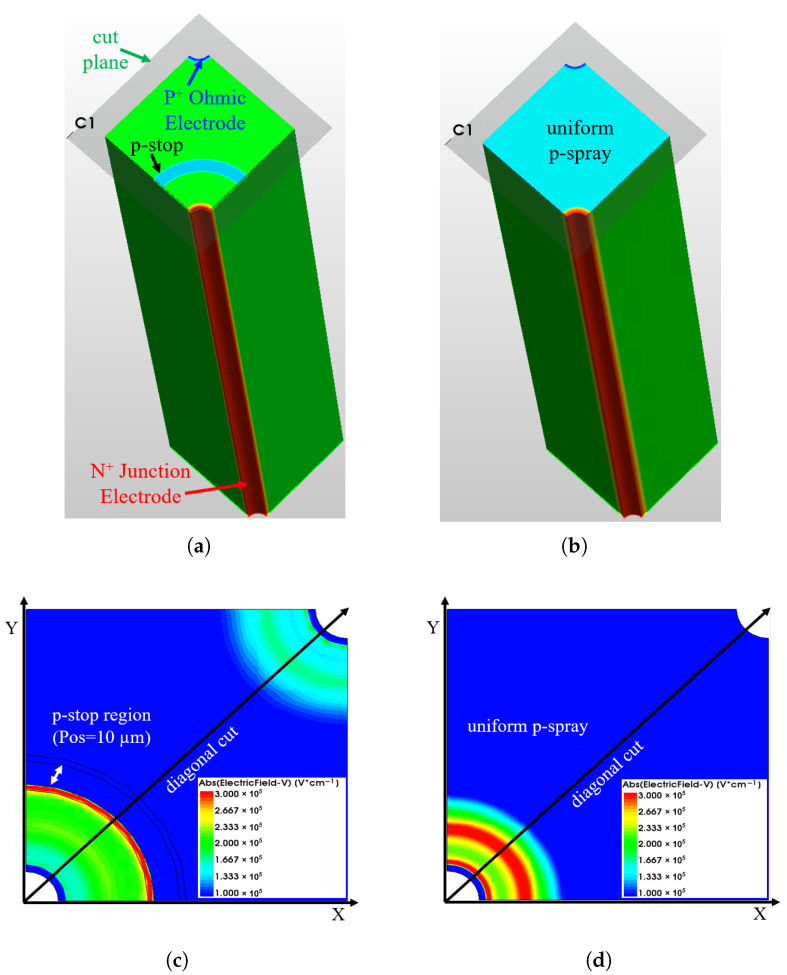
3D simulation domain of different isolation technologies and examples of how the electric fields (before irradiation) are extracted: (**a**) p-stop (with all the configurations of the electrodes and the p-stop indicated); (**b**) p-spray; (**c**) 2D electric field map extracted along the cut plane for the p-stop isolation configuration ($V_{b}$ = 300 V, the cut is made at the depth of the peak p-stop doping concentration); (**d**) 2D electric field map extracted along the cut plane for the p-spray isolation configuration ($V_{b}$ = 130 V, the cut is made at the depth of the peak p-spray doping concentration).

In this paper, TCAD simulations based on Synopsys Sentaurus (Version Q-2019.12) [37] are performed to investigate the limits of the surface isolation layers used in 3D sensors (e.g., 50 × 50−1E) and their impact on the high-voltage tolerance under all relevant irradiation conditions. Figure 2a,b show the three-dimensional TCAD simulation domains which comprise one quarter of a 50 × 50−1E pixel. Utilizing a quarter-pixel sector includes all the features of interest while reducing the simulation time.

However, for convenient evaluation, the current-voltage (I-V) and capacitance–voltage (C-V) curves reported in this work are normalized to the size of a single pixel. Moreover, to highlight where the high electric field regions are located and clarify how the 1D electric field distributions presented in later sections are obtained, Figure 2c,d show examples of 2D electric field maps for both isolation technologies. These maps correspond to the breakdown voltage conditions prior to irradiation.

In the considered devices, the junction electrode has a radius of 2.5 μm. In order to match the layout of the FBK designs, circular field plates extending radially to 6 μm are included in both the ohmic and junction electrodes (not shown), corresponding to an effective field-plate length of 3.5 μm. The $p^{-}$ substrate doping concentration is $10^{12}$ ${\mathrm{cm}}^{-3}$, corresponding to a resistivity on the order of 10 kΩ cm. The doping profiles used in the simulations account for process details provided by FBK (confidential). The junction depths are in the order of 1 μm. The p-stop has a high doping concentration and is designed to enclose the junction electrode in a circular concentric layout (see Figure 1b), similar to that of CNM technology [29], whereas p-spray features a doping concentration reduced by about a factor of 10 and uniformly spread over the entire surface.

The simulation is divided into two parts to study the geometrical effects of the p-stop: position and width. To analyze the impact of the p-stop position on sensor breakdown performance, the width of the p-stop is kept constant (2 μm), while its inner radius is varied from 3.5 μm to 22 μm. Larger radii are not considered since they would cause overlapping of p-stops from neighboring pixels. An analysis of the effect of p-stop width is performed with a fixed p-stop inner radius of 6 μm, 8 μm, 10 μm, or 12 μm, while the width gradually increases, reaching a maximum of 6 μm.

To examine the intrinsic properties of the p-stop without the influence of the junction electrode tip, initial simulations only focus on the core region of the sensor. In order to make the junction electrode structure uniform, its depth has been extended through the entire thickness of the sensor without introducing an oxide layer on the backside. This strategy is also adopted for the study of p-spray. Additional studies incorporating the junction electrode tip have been carried out for both p-stop and p-spray to provide a more comprehensive understanding of the overall sensor performance and to make comparisons with measurements from existing devices.

The simulations use typical models such as the doping-dependent Shockley–Read–Hall generation/recombination model with default parameters, except for the minority carrier lifetimes, which are set to 1 ms to match the FBK technology, and the Van Overstraeten–De Man impact ionization model to simulate breakdown effects [37]. Pre-irradiation scenarios are first studied under room temperature (20 °C) as a baseline. This is followed by the introduction of Perugia models to simulate surface and combined surface-bulk radiation damage, with studies of irradiated sensors carried out at −25 °C. Specifically, in the Perugia Surface Damage model [38], radiation effects are represented by introducing fixed oxide charges (Qox), acceptor-like interface trap states ($D_{it_{\mathrm{acc}}}$), and donor-like interface trap states ($D_{it_{\mathrm{don}}}$). All relevant quantities required for the implementation of the model are provided in [38] (e.g., energy level distribution and electron/hole capture cross-sections). For the sake of clarity, Table 1 summarizes some of the key parameters used in our simulations (with values representative of the FBK technology) before irradiation and after exposure to various TID levels. It is evident that the surface damage effect starts to saturate at a TID of 10–20 Mrad, as also reported in [39]. Hence, in the following simulations, we will consider only TID values of 10 and 20 Mrad. These are also suitable for accounting for surface damage effects caused by background gamma rays in 3D diodes irradiated with reactor neutrons [40], against which our simulations are validated.

The Perugia Bulk Damage model tuned for p-type silicon [41], on the other hand, features deep-level traps (one donor and two acceptor traps) characterized by the energy levels, the respective concentrations that are proportional to the fluence (via introduction rates), and the electron/hole capture cross-sections. In this paper, we consider fluence values from $5.0\times 10^{15}$ to $2.0\times 10^{16}$ $n_{\mathrm{eq}}$ ${\mathrm{cm}}^{-2}$, that well cover the range of interest for 3D sensor applications at the HL-LHC and the range of validity of the available bulk damage model. As an example, Table 2 reports the configuration of the traps in case of a fluence of $1.0\times 10^{16}$ $n_{\mathrm{eq}}$ ${\mathrm{cm}}^{-2}$. While the trap energy levels and associated capture cross-sections remain unchanged, the trap concentrations scale with fluence. Further implementation details of both the surface and bulk damage models can be found in [42].

A comprehensive comparison between simulation results and measurements using FBK devices with p-spray has been the topic of a previous publication [23]. Although there are some discrepancies in the shape of the I-V curves, particularly in the slope of the I-V characteristics at high voltage, it was shown that the Perugia Bulk Damage model can accurately predict the breakdown voltage values after irradiation. This validates the simulation approach and the related models for the present study. Further confirmation is reported in Section 5.

As for the extraction of the breakdown voltage from the I-V curves, similarly to our previous publication [23], we rely on the following formula:

(1) $$k\left(I,V\right)=\frac{\Delta I}{\Delta V}\;\cdot \;\frac{V}{I}\;$$

The breakdown voltage is defined as the value of V for which *k* exceeds a critical value, which is set to 4 here unless otherwise stated. This method allows us to identify changes in the origin of the leakage current (i.e., distinguishing SRH generation from impact ionization effects) in all considered conditions. The method is indeed not sensitive to the value of the leakage current. Nevertheless, the latter must be taken into account to ensure that the estimated breakdown voltage is compatible with the detector system specifications. Among the problems related to a high leakage current are not only the impact on noise but also the power dissipation and the risk of saturation of the readout circuit (ROC). With reference to the considered 3D sensors, an accurate estimate of the leakage current limit, which would result in a degradation of the noise, is difficult because a noise model for the relevant ROCs is not available. The ROC specifications in fact include a maximum leakage current of the detector of 10 nA, which is set to prevent the input amplifier from saturation [43]. Moreover, a maximum power dissipation of 40 mW/${\mathrm{cm}}^{2}$ is set for the 3D pixel detector at a temperature of −25 °C [13]; this limit is, of course, particularly important after severe bulk damage, which increases both the leakage current and the operational voltage. Assuming a fluence of 2.0 × $10^{16}$ $n_{\mathrm{eq}}$ ${\mathrm{cm}}^{-2}$ as representative of the most severe scenario at the HL-LHC, and a maximum operation voltage of ∼150 V (as suggested by beam test results [13,14]), this would correspond to a current limit of ∼0.27 mA/${\mathrm{cm}}^{2}$, that in turn can be scaled to a current limit of ∼6.67 nA for a single pixel of 50 × 50 μ$m^{2}$ area. The latter value (rounded to 6.5 nA for simplicity) is smaller than that set by the first specification (10 nA), and so it can be assumed as the most stringent limit for the current. In spite of the significant increase in the leakage current after bulk damage, due to thermal generation in the bulk, the *k* method with an appropriate critical value allows us to estimate the breakdown voltage at values which are still compatible with a leakage current limit of 6.5 nA, as requested by system specifications. This will be further discussed in Section 5.

## 3. Geometrical Effects of p-Stop Configurations

### 3.1. Influence of p-Stop Position

**Figure 3:**
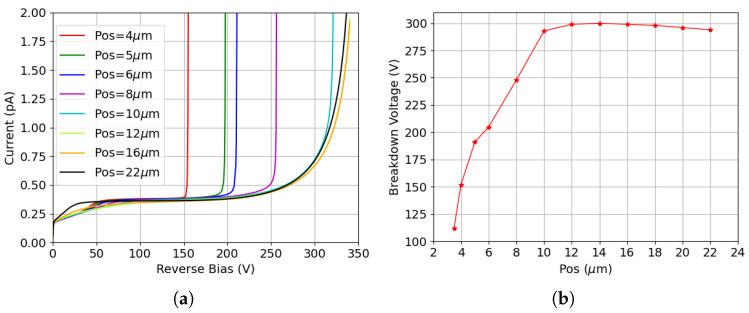
Results for different p-stop positions before irradiation: (**a**) I-V curves for various p-stop configurations (the Pos in the legend refers to the inner radius of the p-stop); (**b**) breakdown voltage for all p-stop positions considered (the X-axis represents the inner radius of the p-stop).

Figure 3a shows the I-V curves before irradiation for different values of the p-stop inner radius, referred to as position (Pos), defined as the distance between the p-stop and the junction electrode. All variations exhibit low leakage current, which mainly originates from thermal generation in the depleted substrate with relatively large generation lifetimes. Additionally, the breakdown voltage increases as the p-stop is positioned farther from the junction electrode before saturating at a maximum p-stop inner radius of 12 μm. However, the breakdown voltage eventually decreases when the p-stop is placed too close to the ohmic electrode (e.g., Pos = 22 μm). This relationship is made more evident in Figure 3b, which reports the breakdown voltage as a function of p-stop position. The lowest breakdown voltage is obtained when the p-stop is positioned closest to the junction electrode with an inner radius of 3.5 μm. Despite being fully covered by the field plate in this configuration, the highly doped p-stop is in close proximity to the junction electrode. This gives rise to an electric field peak high enough to cause breakdown at a voltage on the order of 100 V. As the p-stop moves away from the junction electrode, the breakdown voltage rapidly increases before saturating at a distance of 12 μm. A decrease is again visible when the radius becomes very large, and the p-stop approaches the ohmic electrode.

**Figure 4:**
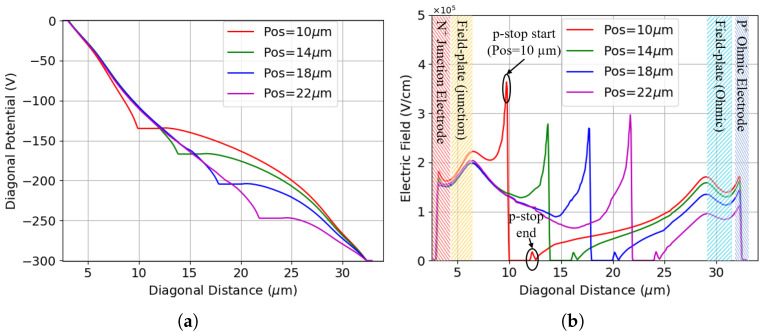
Results for a few selected p-stop positions at $V_{b}$ = 300 V (the cut depth is at the peak p-stop doping concentration): (**a**) the potential profile; (**b**) the electric field profile (Despite the junction/ohmic field plate extending from the respective electrode to its full designed length, the shaded regions representing the electrodes and the field plates are made to not overlap each other for better visualization. This approach is consistently applied across all relevant plots presented subsequently).

It has been observed that across all considered positions, the breakdown originates from the inner side of the p-stop. Figure 4 shows the electric potential and electric field profiles for a few selected p-stop positions as a function of distance from the junction electrode. These profiles are obtained by first slicing the sensor along a plane parallel to the surface at the depth of the peak p-stop doping concentration (see the cut plane indicated in Figure 2a). A cut is then applied perpendicular to the surface progressing diagonally from the junction electrode to the ohmic electrode (see the diagonal cut indicated in Figure 2c). Different radii ranging from 10 μm to 22 μm are evaluated at the same bias potential of 300 V, which is close to the maximum breakdown voltage, to allow direct appreciation of the influence of p-stop positions on the breakdown voltage. Figure 4a shows that as the position increases and the p-stop is brought closer to the p-type column, the p-stop potential grows negatively. Because the slope of the potential determines the electric field, kinks in the potential at the p-stop inner radius cause a peak in the electric field distribution. Figure 4b shows the electric field distribution as a function of distance from the junction electrode, with different shading used to indicate the distinct regions. The maximum electric field is obtained at a p-stop inner radius of 10 μm, causing this configuration to have the lowest breakdown voltage among the four cases. The electric field peak decreases as the radius increases, therefore delaying the breakdown. The field begins to increase again when the radius reaches 22 μm, resulting in a slightly lower breakdown voltage.

In all cases, sizable secondary electric field peaks are present at the junction electrode, the ohmic electrode, and the edges of their field plates. In particular, the electric field peak at the ohmic electrode arises from the relatively large voltage drop between the $p^{+}$ doping at the ohmic column opening and the layer of electrons at the surface induced by the positive oxide charge. Since the related electric field peak is much lower than that at the inner side of the p-stop, this region is not responsible for the breakdown before irradiation, but it strongly affects the breakdown performance after surface damage, when the increase in the positive oxide charge density makes the concentration of the electrons in the surface layer much larger.

**Figure 5:**
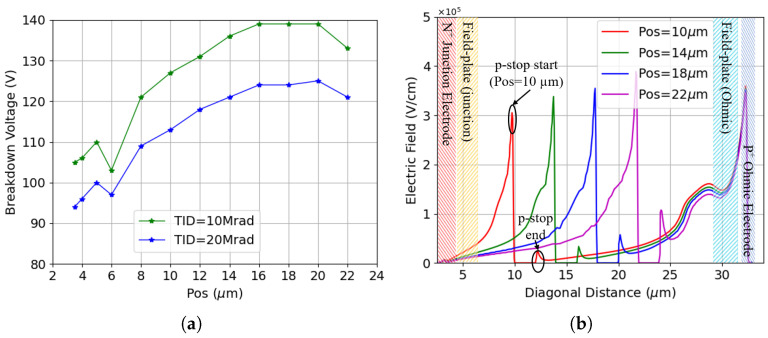
Results for different p-stop positions after surface damage: (**a**) breakdown voltage; (**b**) electric field for a few selected p-stop positions at $V_{b}$ = 120 V, with a TID of 10 Mrad.

Figure 5a shows the breakdown voltage and electric field profile after surface damage. Total ionizing doses (TIDs) of 10 and 20 Mrad have been considered, both showing substantially decreased breakdown voltages compared to pre-irradiation cases. However, the trend remains the same. Positioning the p-stop further away from the junction electrode increases the breakdown voltage until the inner radius becomes too large, and the p-stop approaches the ohmic electrode. The dips in breakdown voltage observed at a p-stop inner radius of 6 μm can be attributed to the fact that this exactly corresponds to the edge of the field plate extending from the junction electrode.

Distinct from pre-irradiation conditions where a dominant electric field peak appears on the inner side of the p-stop, the electric field distribution for irradiated sensors is quite different, as seen in Figure 5b. This effect is because of the increased fixed oxide charge density induced by surface damage. To examine the behavior after irradiation, a bias potential of 120 V is used to reconstruct the electric field due to the decreased breakdown voltage; the TID corresponds to 10 Mrad. Under these conditions, two primary electric field peaks are observed: one on the inner side of the p-stop, which increases as the p-stop moves closer to the ohmic electrode, and another on the ohmic electrode, which remains stably high and independent of the p-stop position. Although the electric field peaks in all cases are high enough to already cause an avalanche at 120 V, the breakdown voltage is delayed for more than 10 V thanks to the strong surface trapping effects [44]. A comparison of Figure 4b and Figure 5b shows the absence of electric field peaks at the edge of the junction electrode field plate after surface damage, leading to the conclusion that the field plate is not effective in enhancing the breakdown voltage. In fact, the field plate is at the same junction electrode potential, which is more positive than the substrate potential. In this condition, the field plate acts as a gate electrode of a MOS capacitor, which inverts the surface underneath. On the contrary, after surface damage, the fixed oxide charge density is high enough to lead the surface to inversion regardless of the presence of the field plate.

**Figure 6:**
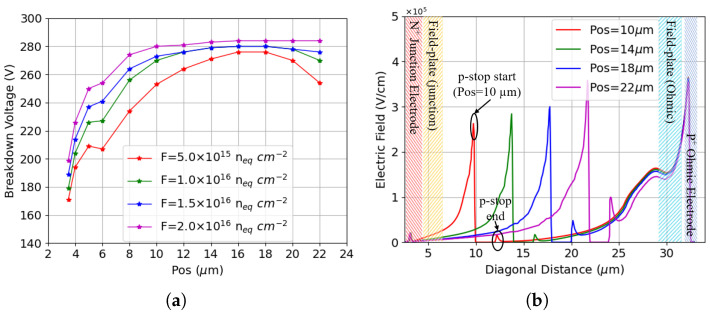
Results for different p-stop positions with combined radiation damage: (**a**) breakdown voltage at TID = 10 Mrad for different fluences; (**b**) electric field for a few selected p-stop positions at $V_{b}$ = 120 V, TID = 10 Mrad and F = 1.0 × $10^{16}$ $n_{\mathrm{eq}}$ ${\mathrm{cm}}^{-2}$.

The combined radiation damage effect is studied by adding the bulk damage model on top of the surface damage model. Figure 6a shows the results of different levels of bulk damage with a TID of 10 Mrad. It is evident that the breakdown performance can be greatly recovered in the presence of bulk damage. As in pre-irradiation cases, the breakdown voltage saturates as the p-stop inner radius gets larger. A small increase in the breakdown voltage with increasing fluence is also observed. With the exception of slightly reduced breakdown values, the behavior is the same using a TID of 20 Mrad (not shown).

**Figure 7:**
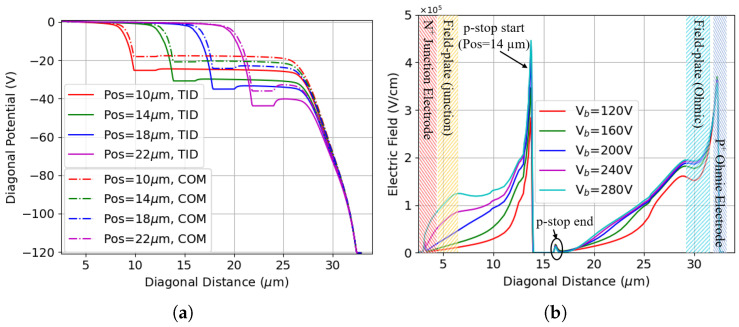
Results of different radiation damages: (**a**) the potential difference between surface damage (TID) and combined radiation damage (COM), the reverse bias applied is 120 V; (**b**) electric field with varying reverse biases, the p-stop is positioned at 14 μm, the TID is 10 Mrad, and the Fluence is 1 × $10^{16}$ $n_{\mathrm{eq}}$ ${\mathrm{cm}}^{-2}$.

Figure 6b shows the electric field generated by a bias of 120 V after a fluence of 1 × $10^{16}$ $n_{\mathrm{eq}}$ ${\mathrm{cm}}^{-2}$. Similar to the surface damage case, there are two main electric field peaks: one on the inner side of the p-stop and the other on the ohmic electrode. While the latter is not sensitive to the p-stop position, the former increases with the inner radius, a behavior analogous to when only surface damage is present. The electric field profiles and the peak values for the combined case are similar to those of Figure 5b. However, Figure 6a shows that the breakdown voltage values increase significantly after the introduction of bulk damage. To understand the mechanism behind this behavior, the potential is compared across the same 2D plane in all cases with surface damage alone and with combined radiation damage. Figure 7a shows the diagonal potential in both cases under a bias potential of 120 V. The TID considered is 10 Mrad with a fluence of 1 × $10^{16}$ $n_{\mathrm{eq}}$ ${\mathrm{cm}}^{-2}$. The potential drop between the p-stop and the junction electrode decreases across all configurations in response to the increased effective doping concentration induced by bulk radiation damage [45]. While this partially accounts for the improved breakdown voltage after bulk damage, it does not entirely justify the largely enhanced performance because the peak electric field values at 120 V already approach those required to trigger avalanche breakdown. For example, Figure 7b shows the evolution of the electric field with bias voltage for the p-stop positioned at 14 μm which has a breakdown voltage of ∼280 V. Despite the high electric field peak near the ohmic electrode, avalanche breakdown does not occur until the electric field peak at the inner radius of the p-stop reaches the critical value at a much larger bias voltage. The reason for this behavior can be mainly attributed to the strong trapping effect after bulk damage: radiation-induced defects in the bulk act as scattering centers for the energetic carriers responsible for the impact ionization phenomenon, thus reducing their mean free path, which results in a quenching mechanism on the avalanche [46].

The breakdown performance degradation of p-stop-equipped sensors after surface damage suggests the need for further optimization. Figure 5b shows that the electric field peak at the inner p-stop radius can be tuned by carefully adjusting the p-stop position so that the second electric field peak close to the ohmic electrode becomes the primary cause of avalanche breakdown. Therefore, further improvements to the electric field distribution at the p-stop, e.g., by equipping it with a field plate as proposed in [47], would be ineffective besides adding technological complexity. However, the electric field peak at the ohmic electrode can be mitigated by extending the length of the poly-Si field plate. This is a simple solution that does not require any extra lithography steps.

**Figure 8:**
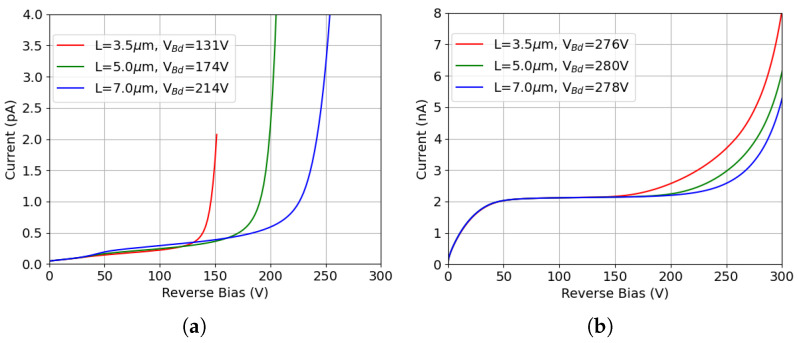
I-V curves for different ohmic field-plate lengths (all the results are obtained with the p-stop positioned at 12 μm and a width of 2 μm, L = 3.5 μm represents the default field-plate length on the ohmic electrode used in all the simulations summarized above): (**a**) only surface damage (TID = 10 Mrad); (**b**) combined radiation damage (TID = 10 Mrad, F = 1 × $10^{16}$ $n_{\mathrm{eq}}$ ${\mathrm{cm}}^{-2}$). The related breakdown voltages are reported in the legend.

Figure 8 shows the I-V curves for different ohmic field-plate lengths. All results are obtained with the p-stop positioned at 12 μm (with the inner radius set accordingly) and a width of 2 μm. The default field-plate length L = 3.5 μm on the ohmic electrode is used in all the simulations summarized above. As anticipated, the breakdown voltage sizably increases with increasing field-plate length, exceeding 210 V with a field-plate length of 7 μm after the same level of TID (10 Mrad). Extending the field plate does not result in adverse effects on the breakdown performance of the sensor after bulk damage, as displayed in Figure 8b, further demonstrating the effectiveness of the proposed solution.

### 3.2. Influence of p-Stop Width

In addition to the position, the p-stop width plays a key role in the breakdown performance of the sensor as well. The influence of p-stop width has been studied by maintaining a constant p-stop inner radius while increasing the width gradually from 2 μm to 6 μm. The analysis is then repeated with different p-stop positions.

**Figure 9:**
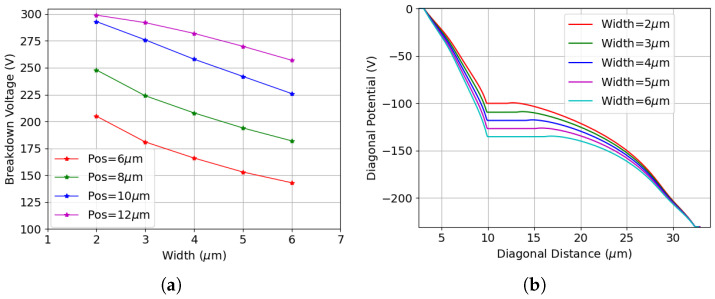
Results of different p-stop widths before irradiation (different p-stop positions are also reported): (**a**) breakdown voltage; (**b**) potential for different p-stop widths at $V_{b}$ = 230 V, p-stop inner radius is 10 μm.

Figure 9a summarizes the breakdown voltage of all the configurations investigated: different widths and positions. The breakdown voltage increases as the p-stop gets farther from the junction electrode for all p-stop widths, in line with previous observations. Conversely, it decreases as the p-stop width increases. This can be explained by looking at Figure 9b, which reports the potential across the p-stop positioned at 10 μm with different widths with a constant reverse bias. It is evident that the potential drop between the p-stop and junction electrode increases as the p-stop gets wider, a pattern matching what was reported in [34]. As a result, the electric field (not shown here) shows that the wider the p-stop gets, the higher the electric field peak is, which leads to lower breakdown voltages.

**Figure 10:**
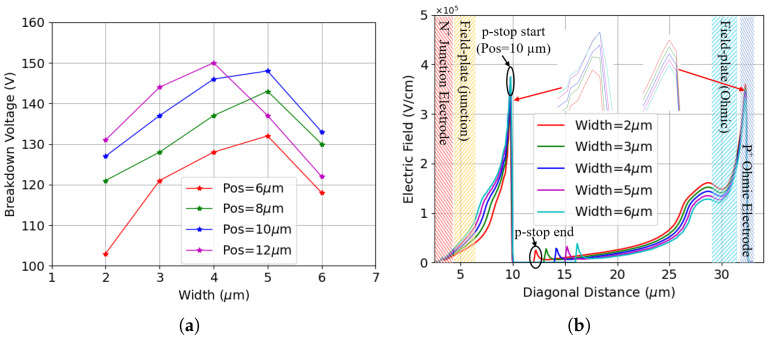
Results of different p-stop widths after surface damage (TID = 10 Mrad): (**a**) breakdown voltage for different values of the inner radius; (**b**) electric field for different p-stop widths at $V_{b}$ = 120 V, p-stop inner radius is 10 μm.

The breakdown behavior after surface damage has more complex patterns, as depicted in Figure 10a. Generally, a higher breakdown voltage is observed as the p-stop is moved away from the junction electrode, similar to the pre-irradiation cases. The breakdown voltages initially increase as the p-stop width is increased, but they decrease if the p-stop is too wide. Figure 10b illustrates the electric field for various p-stop widths at $V_{b}$ = 120 V, with a p-stop inner radius fixed at 10 μm. The two main electric field peaks have comparable values, but, as illustrated in the subplots, their dependence on the p-stop width is opposite. The first peak decreases as the p-stop width decreases, while the second peak is weakest at the largest p-stop width and increases as the width reduces. Nonetheless, both peaks can reach critical values, and the breakdown performance is highly dependent on the p-stop configuration. In this case, the highest breakdown voltage is achieved when the p-stop width is 5 μm.

**Figure 11:**
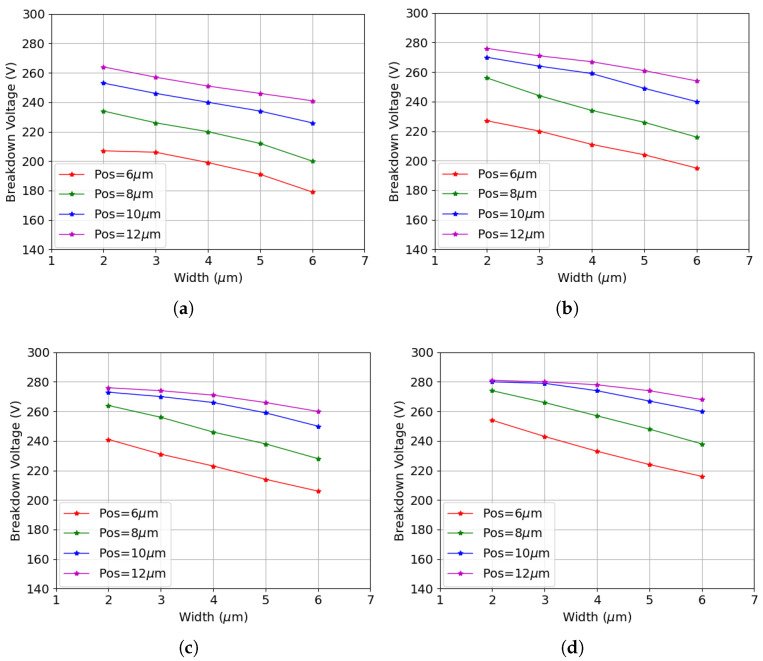
Results for different p-stop widths after combined radiation damage at TID = 10 Mrad and different fluences: (**a**) F = 5.0 × $10^{15}$ $n_{\mathrm{eq}}$ ${\mathrm{cm}}^{-2}$; (**b**) F = 1.0 × $10^{16}$ $n_{\mathrm{eq}}$ ${\mathrm{cm}}^{-2}$; (**c**) F = 1.5 × $10^{16}$ $n_{\mathrm{eq}}$ ${\mathrm{cm}}^{-2}$; (**d**) F = 2.0 × $10^{16}$ $n_{\mathrm{eq}}$ ${\mathrm{cm}}^{-2}$.

The non-monotonic pattern disappears after the introduction of bulk damage, as shown in Figure 11. Likewise, four different fluences are considered, with the highest value reaching 2 × $10^{16}$ $n_{\mathrm{eq}}$ ${\mathrm{cm}}^{-2}$. The qualitative trend is the same as before irradiation: the breakdown voltage increases as the p-stop moves farther from the junction electrode, but decreases with increasing p-stop width. The significant concentration of trapping centers introduced by bulk damage (see e.g., Table 2) introduces a strong quenching effect on the avalanche [46], which helps counteract the adverse effects of surface damage. As a result, the breakdown voltage substantially increases, with values approaching those obtained before irradiation.

## 4. Characteristics of p-Spray Isolation

**Figure 12:**
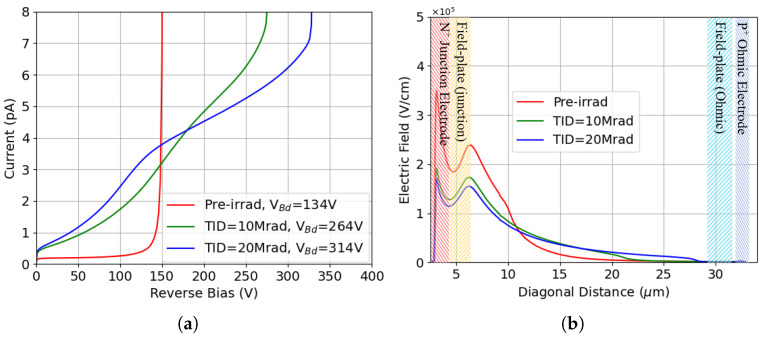
Results of p-spray isolation: (**a**) I-V curves before irradiation and after surface damage; (**b**) electric field at $V_{b}$ = 130 V.

The intrinsic properties of p-spray isolation have been explored with the same approach. Since p-spray introduces a uniform doping everywhere, there’s a direct contact between the p-spray layer and the junction electrode, so that the breakdown voltage before irradiation (∼130 V) is lower than that of p-stop, as shown in Figure 12a. Regardless, the advantage of this isolation technique is evident: the breakdown voltage sizably increases after surface damage. This is mainly due to the increased oxide charge density, which partially compensates the effect of the p-spray doping, thus reducing the electric field peak at the junction between the p-spray and $n^{+}$ electrode. This is evident from Figure 12b, where the effectiveness of the field plate in splitting the main electric field peak into two sub-peaks, with a beneficial effect on the breakdown voltage, can also be appreciated, especially after surface damage.

**Figure 13:**
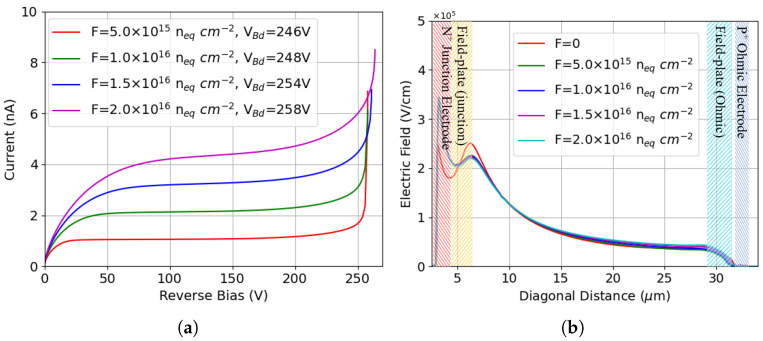
Results of p-spray isolation: (**a**) I-V curves after combined radiation damage (TID = 10 Mrad); (**b**) corresponding electric field at $V_{b}$ = 240 V, compared to that without bulk damage (F = 0 in the legend) at the same TID and voltage.

Figure 13a shows the I-V curves after different levels of bulk damage, with the TID kept at 10 Mrad. In the presence of bulk damage, the leakage current increases by orders of magnitude due to the much stronger contribution from thermal generation in the bulk. The simulated values, in the order of a few nA, are in good agreement with measurements [23] and with analytical predictions based on the current damage constant $\alpha ≃4\times 10^{-17}$ A/cm [45]. It can be seen that the breakdown voltage, which can still be reliably extracted with the *k* method, slightly decreases with respect to the case of surface damage alone at the same TID. Bulk damage has a combined action on the effective doping concentration and on charge trapping. In case of p-spray, the enhanced charge trapping and related reduced carrier mean free path do not play a dominant role due to the very close proximity between the high-field region and the junction electrode. On the contrary, as can be observed in Figure 13b, the addition of bulk damage, regardless of the fluence, makes the two electric field peaks less balanced, with a more pronounced peak at the junction electrode, which justifies a slight decrease in the breakdown voltage.

## 5. Experimental Validation of Simulation Results

### 5.1. Simulations Including the Effect of Column Tips

During fabrication of small-pitch 3D sensors, junction electrodes are kept at a safe distance from the highly doped support wafer at the backside. This introduces tips at the bottom of the electrodes due to the nature of the DRIE process, illustrated in Figure 1a. These tips lead to electric field focusing and cause a risk of premature breakdown. To analyze the influence of the tip on the breakdown voltage in simulation, a tip is included on the junction electrode [23]. Motivated by previous simulations, the p-stop used in this study has a width of 2 μm and an inner radius of 12 μm.

**Figure 14:**
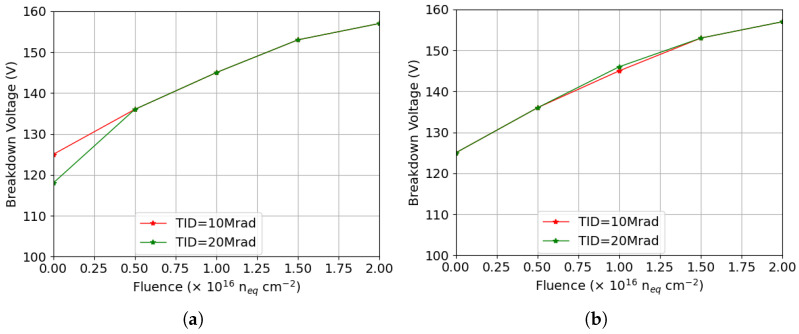
Breakdown voltages extracted from simulation results as a function of bulk damage fluence at two TID values with the presence of the tip: (**a**) p-stop isolation (p-stop position and width set to 12 μm and 2 μm, respectively); (**b**) p-spray isolation.

The breakdown voltage obtained before irradiation for this configuration is 129 V, much smaller than that previously obtained with passing-through junction electrodes, suggesting that the breakdown originates from the tip. Figure 14a shows the results of the structure after radiation damage, where Fluence = 0 indicates that only surface damage is present. The calculated breakdown voltage is 125 V when the TID is set to 10 Mrad and bulk damage is absent, which is slightly lower than the 131 V of the passing-through structure in this condition. Therefore, the breakdown is likely a combined effect on both the surface and tip, whereas at TID = 20 Mrad, the breakdown voltage decreases, indicating that the p-stop is responsible. Most importantly, the breakdown voltage does not improve after bulk damage to the same extent as observed with the passing-through electrode, proving that the tip is the reason for the breakdown.

Figure 14b shows the corresponding results for p-spray isolation. The same breakdown voltages were obtained with different levels of surface damage, and they are significantly smaller than those of the structure without the tip, confirming that the tip defines the upper limit of the breakdown voltage for this isolation technology after surface damage. Moreover, it is evident that both structures (i.e., p-stop and p-spray) exhibit the same breakdown voltage after combined radiation damage, as the source of the breakdown lies at the tip.

### 5.2. Comparison with Experimental Results

In order to assess the validity of the simulation approach and the radiation damage models employed in this study, the simulated results are compared with the measured data reported in [13,48].

In the presence of bulk damage, besides a considerable increase in the leakage current due to thermal generation in the bulk [45], the experimental I-V characteristics exhibit a more gradual increase at large voltage than the simulated ones [23]. This makes the original critical *k* value of 4 problematic because it would lead to extremely large breakdown voltages, which would be incompatible with the current limit (6.5 nA) set by the system specifications (see Section 2). To address this problem, the critical value of *k* was adjusted to 2.5, allowing for a more reliable estimation of breakdown voltages from the experimental curves. The corresponding breakdown voltage values are, in fact, compatible with the maximum power dissipation density, as also shown in [13]. The simulated I-V curves, on the other hand, exhibit a sharper slope at breakdown, making the extracted breakdown voltage largely insensitive to the choice of the critical *k*. This behavior also holds in simulations for structures without the tip; although they yield slightly lower absolute breakdown voltages with the updated *k*, the overall trends discussed earlier remain unaffected.

While the detailed layout of the p-stop (specifically its position and width) in the CNM sensors is not explicitly known, the breakdown characteristics are predominantly influenced by the geometry of the junction electrode tip, both before irradiation and after exposure to combined radiation effects, as outlined earlier. To enable a more representative comparison with the CNM sensors, simulations were repeated implementing an electrode radius (r) of 4 μm, which reflects their actual design [13,48]. All the other geometrical parameters were kept consistent: a p-stop width of 2 μm, an inner radius of 12 μm, and an effective field plate length of 3.5 μm.

The experimental breakdown voltage values extracted for the two isolation technologies in different conditions are summarized in Table 3, along with the corresponding simulation results. As evident from the comparison with the breakdown voltage values reported in the previous Sections and the inspection of the electric field plots, the breakdown occurs at the column tip in all cases. For structures with p-stop, the measured breakdown voltage is 127 ± 14 V before irradiation, closely matching the simulated result of 138 V. The simulation also aligns well with measurements at a fluence of 1.0 × $10^{16}$ $n_{\mathrm{eq}}$ ${\mathrm{cm}}^{-2}$. The agreement is less satisfactory when the fluence is 1.5 × $10^{16}$ $n_{\mathrm{eq}}$ ${\mathrm{cm}}^{-2}$, but it is still reasonably good considering the several uncertainties on the layout and process details for CNM sensors, in particular the actual junction electrode depth. In comparison, the breakdown voltages for the p-spray configurations show strong consistency across all tested conditions, further proving the robustness of the radiation damage models used. Although further refinement of the damage models is necessary to more accurately capture the detailed shape of post-irradiation I-V curves, the current framework provides reliable predictions of the breakdown behavior for both isolation technologies.

## 6. Impact of Different Isolations on the Capacitance

In addition to their diverse responses to radiation damage in terms of breakdown performance, the two isolation technologies also affect the sensor’s capacitance differently, with possible impact on the noise. In planar sensors, this effect is particularly pronounced in the interstrip (interpixel) capacitance [34]. On the contrary, in 3D sensors, the interpixel capacitance is negligible with respect to the bulk capacitance [49]. Therefore, in the following, we will limit the analysis to the bulk component of the capacitance.

**Figure 15:**
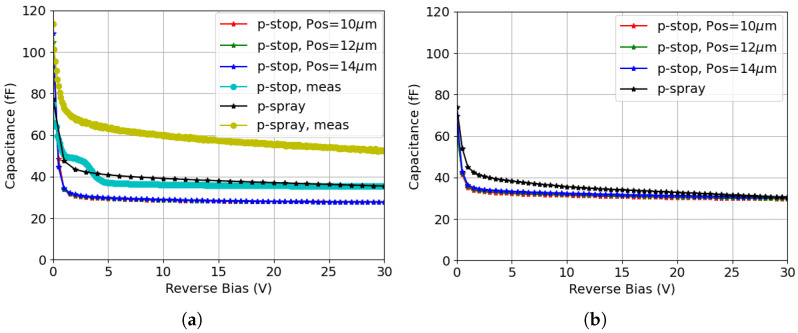
Capacitance–voltage curves for different isolation techniques: (**a**) before irradiation (simulation and measurement results are indicated in the legend with different labels); (**b**) after surface damage (based on simulation, TID = 10 Mrad).

Figure 15a compares the simulated and measured capacitance prior to irradiation for structures representative of CNM and FBK technologies. The signal frequency used for C-V measurements and simulations is 1 kHz. Various p-stop configurations—specifically different positions with a fixed width of 2 μm—are explored to assess their influence on the capacitance. Resulting from the short inter-electrode distance characteristic of small-pitch 3D pixel sensors, full depletion can be achieved with only a few volts. Hence, the maximum voltage applied during the measurements was limited to 30 V.

The simulation shows that both structures experience a sharp decrease in capacitance as the bias increases, on account of the rapid depletion of the active volume. The p-spray structure has a higher capacitance at full depletion owing to the direct contact between the p-spray layer and the junction electrode. In contrast, the p-stop configuration separates the surface region into two distinct parts and connects them in a series capacitance arrangement, resulting in a lower capacitance. The difference between the various p-stop configurations, however, is negligible. Noticeable offsets between the simulated and measured capacitance are observed for both isolation types. These offsets arise from the measurements being performed on diodes [13] that have metal grids running on top of the passivation layer to short all pixels together. These grids, along with relatively large metal probing pads, introduce an additional large capacitance. From measurements on MOS capacitors from FBK batches with p-spray, this capacitance was estimated to contribute about 18 fF per pixel, which well matches the observed offset between the simulated and measured curves. For CNM sensors, an exact estimation of the grid contribution to the capacitance is not possible since the layout and process details are not available. However, consistent with the observed offset, it is expected to be slightly smaller because of the absence of p-spray, leading to a smaller capacitance in the inversion condition. The presence of the metal grid + pad is also believed to be responsible for the bump observed in the p-stop experimental curves at a reverse bias of ≃2.5 V. A bump is indeed also present in the measurement for p-spray due to the same reason, but it occurs at voltages larger than 30 V due to the much larger doping concentration of p-spray with respect to the $p^{-}$ substrate (an analytical estimate of the flat-band voltage in the two situations and a direct measurement on a MOS capacitor in case of p-spray, return values in good agreement with the observed positions of the bumps).

Comparable results are instead obtained in the two structures after surface damage, as shown in Figure 15b, with an initial rapid decrease with bias. At full depletion, the capacitance of the p-spray structure is lower than before irradiation as a result of the increased fixed oxide charge density, which expands the surface depletion region. In comparison, the capacitance of the p-stop structure slightly increases after surface damage, because of the increased concentration of the electron inversion layer induced by the increased oxide charge density. However, the two isolation techniques have the same capacitance after surface damage at the considered TID.

**Figure 16:**
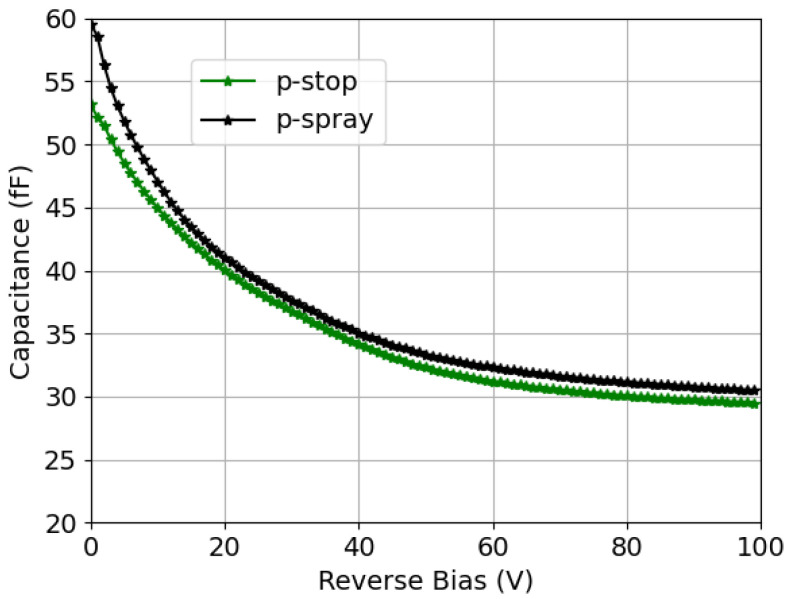
Simulated capacitance–voltage curve for the both structures after combined radiation damage, the TID is set to 10 Mrad and the fluence considered is 1.0 × $10^{16}$ $n_{\mathrm{eq}}$ ${\mathrm{cm}}^{-2}$ (in the p-stop structure, the p-stop Pos is set to 12 μm).

To better understand the impact of surface + bulk radiation damage on the sensor capacitance for both isolation structures, simulations are performed incorporating the full combined radiation damage model. Similarly, the frequency is set to 1 kHz, and the fluence considered is 1.0 × $10^{16}$ $n_{\mathrm{eq}}$ ${\mathrm{cm}}^{-2}$. As shown in Figure 16, the capacitance exhibits a relatively smooth decrease before saturating at roughly the same minimum value in both structures. The delayed decrease in the capacitance with respect to Figure 15b is attributed to the fact that much higher reverse bias voltages are needed to fully deplete the active volume after bulk damage. Regardless of this, the two curves are very similar and their saturation values align closely with those obtained from previous simulations.

## 7. Conclusions and Outlook

This work investigates, for the first time with the aid of TCAD simulations, the impact of different surface isolation technologies on the high-voltage tolerance of 3D sensors. In order to focus on the intrinsic impact of the isolation layers, simplified pixel structures are initially simulated, where the backside does not introduce further complexity. Relevant values of the breakdown voltages for both technologies are summarized in Table 4. A fluence of 1.0 × $10^{16}$ $n_{\mathrm{eq}}$ ${\mathrm{cm}}^{-2}$ is here considered as representative of the surface + bulk damage.

The breakdown voltage of sensors using the p-stop method before irradiation is positively correlated with the distance from the junction electrode, reaching saturation at a distance of ∼12 μm and degrading again when approaching the ohmic electrode. This suggests suboptimal performance of the CNM design with the p-stop positioned close to the junction electrode [29], whereas the layout reported in [48] is expected to yield superior results. There is indeed an interval of p-stop positions, from ∼12 μm to ∼20 μm, for which the breakdown voltage is maximum. Any p-stop position within this optimal range can be chosen; it can also be chosen according to the needs of a real layout implementation. As an example, the p-stop in the layout of Figure 1b has a position of 12 μm, but a larger one could be used in order to avoid overlap with the n-poly Si layer.

Additionally, the breakdown voltage decreases with increasing p-stop width. This negative correlation necessitates the use of a narrow p-stop, provided that it complies with layout rules. This, of course, depends on the specific fabrication process and especially on the lithographic equipment used. In every instance, the performance of sensors using p-stop suffers from serious deterioration of the breakdown voltage after surface damage, primarily due to the electric field peak at the ohmic electrode. Our simulations suggest that a partial improvement could be obtained by extending the field-plate length at the ohmic electrode. As long as the pixel layout is not too dense, which could prevent us from using longer field plates, this proposed solution can be readily applied without adding any process complexity since it only requires a simple layout modification to the p-poly Si mask. Regardless of the field-plate length, very high breakdown voltage values can be recovered after bulk damage as a consequence of trapping effects, which quench the avalanche phenomenon.

Conversely, p-spray leads to lower breakdown voltages before irradiation due to the direct contact of the p-spray layer and the junction electrode. The breakdown voltage is largely improved after surface damage and remains high after bulk damage.

Further simulations have been performed to evaluate the sensor capacitance using different isolation techniques, with results showing that p-stop may lead to a lower capacitance compared to p-spray before irradiation, with little sensitivity to the p-stop position. However, the difference becomes negligible after surface damage. The difference also remains negligible after adding bulk damage, in spite of the significant changes in the C-V curves at low voltage due to delayed depletion of the substrate.

The overall performance comparison between p-stop and p-spray does not show clear indications favoring one technique over the other. The superior breakdown performance of p-stop (before irradiation) and p-spray (after surface damage) is irrelevant for practical uses. Before irradiation, the required bias voltage is much lower than the worst-case breakdown voltage, thus ensuring wide operational margins for both isolation techniques. Moreover, there are as yet no significant applications of 3D sensors that involve only surface damage (e.g., with X-rays), so the degraded breakdown performance of p-stop in this condition is not a serious concern. While p-stop ensures a lower capacitance before irradiation, hence lower noise, signals are always much larger than noise under this condition, so this advantage of p-stop over p-spray is also not fundamental.

Succinctly, a practical reason why p-spray should be preferred is that is does not require a lithographic step, thus easing the fabrication process, reducing costs, and improving yield, as most defects are related to lithography. This advantage will become even more important if the pixel pitch is further reduced, as expected by the current trend in HEP applications. In fact, very small-pitch (∼30 μm) 3D pixels will have a very dense layout with more severe constraints on the p-stop placement.

To evaluate the sensors’ breakdown performance in a more realistic way, further studies have been carried out by adding a tip to the bottom of the junction electrode. This is the solution currently adopted by all foundries for small-pitch 3D sensors. Simulation results show that the high electric field focusing on the junction column tip limits the sensors from reaching very high operation voltages, regardless of the surface isolation technique, which is in good agreement with measurements on existing samples irradiated to HL-LHC fluences.

As long as the pixel size is not too small, this shortcoming could be attenuated by increasing the radius of the junction electrode, leading to a breakdown voltage improvement of a few tens of Volts [23]. For very small pixels, where increasing the column radius would not be feasible, a solution could consist in ending the column etching step isotropically, so as to round the tip and locally increase its radius [50]. Alternatively, a different technological approach could be pursued with junction electrodes passing entirely through the bulk. SOI wafers should be used in order to make this solution compatible with the present single-sided processes with backside bias. Ohmic electrodes could be etched through the bonding oxide to reach the highly doped handle wafer, as demonstrated in [51]. However, fully penetrating junction electrodes would require the addition of isolation layers on the backside, which would then become the most critical regions for breakdown, calling for a dedicated optimization study. Regardless of the technological approach, future work beyond the current study should consider the requirements of next-generation high-energy particle colliders in terms of smaller pixel sizes and/or increased radiation hardness. To this latter end, presently available radiation damage models, which have so far been validated up to fluences of the order of ∼$10^{16}$ $n_{\mathrm{eq}}$ ${\mathrm{cm}}^{-2}$, are being extended to fluences exceeding ∼$10^{17}$ $n_{\mathrm{eq}}$ ${\mathrm{cm}}^{-2}$. We are contributing to this effort, in collaboration with the University of Perugia, through the characterization of test structures and the validation of modified models by 3D sensor simulations.

## Figures and Tables

**Table 1:** Fixed oxide charge ($Q_{\mathrm{ox}}$) and interface trap densities ($D_{it}$) calculated according to the Perugia model [38] with various TID levels.

TID	$Q_{\mathrm{ox}}$ (${\mathrm{cm}}^{-2}$)	$D_{{\mathrm{it}}_{\mathrm{acc}}}$ (${\mathrm{eV}}^{-1}$${\mathrm{cm}}^{-2}$)	$D_{{\mathrm{it}}_{\mathrm{don}}}$ (${\mathrm{eV}}^{-1}$${\mathrm{cm}}^{-2}$)
Pre-irradiation	$8.0\times 10^{10}$	$2.33\times 10^{10}$	$2.33\times 10^{10}$
10 Mrad	$9.52\times 10^{11}$	$4.38\times 10^{12}$	$1.82\times 10^{12}$
20 Mrad	$1.04\times 10^{12}$	$4.85\times 10^{12}$	$2.05\times 10^{12}$
100 Mrad	$1.24\times 10^{12}$	$5.93\times 10^{12}$	$2.58\times 10^{12}$

**Table 2:** Configuration of the traps after a fluence of $1.0\times 10^{16}$ $n_{\mathrm{eq}}$ ${\mathrm{cm}}^{-2}$.

Trap Type	Energy (eV)	Concentration (${\mathrm{cm}}^{-3}$)	$\sigma_{e}$ (${\mathrm{cm}}^{2}$)	$\sigma_{h}$ (${\mathrm{cm}}^{2}$)
Donor	$E_{c}$ − 0.23	$6\times 10^{13}$	$2.3\times 10^{-14}$	$2.3\times 10^{-15}$
Acceptor 1	$E_{c}$ − 0.42	$1.613\times 10^{16}$	$1.0\times 10^{-15}$	$1.0\times 10^{-14}$
Acceptor 2	$E_{c}$ − 0.46	$9\times 10^{15}$	$7.0\times 10^{-15}$	$7.0\times 10^{-14}$

**Table 3:** Breakdown voltages (extracted with k = 2.5) under various operating conditions. Simulation results (sim) are compared to measurements (meas) for both p-stop and p-spray. For measurements, average values ± standard deviations of results from different samples are reported. For all the considered fluences, the simulation used a TID of 10 Mrad.

	p-Stop, Meas (r = 4.0 μm)	p-Stop, Sim (r = 4.0 μm)	p-Spray, Meas (r = 2.5 μm)	p-Spray, Sim (r = 2.5 μm)
Pre-irradiation	127 ± 14 V [48]	138 V	128 ± 8 V [13]	125 V
F = 1.0 × $10^{16}$ $n_{\mathrm{eq}}$ ${\mathrm{cm}}^{-2}$	173 ± 12 V [13]	169 V	133 ± 16 V [13]	145 V
F = 1.5 × $10^{16}$ $n_{\mathrm{eq}}$ ${\mathrm{cm}}^{-2}$	206 ± 13 V [13]	180 V	145 ± 7 V [13]	153 V

**Table 4:** Simulated intrinsic breakdown voltages for p-stop and p-spray under various operating conditions. Values for p-stop refer to different positions and widths, with a standard field plate length. For surface damage, a TID of 10 Mrad is considered; for surface + bulk damage, the same TID is used with an additional fluence of $10^{16}$ $n_{\mathrm{eq}}$ ${\mathrm{cm}}^{-2}$.

Isolation Configuration	Radiation Condition	Width (Only for p-Stop)				
		2 μm	3 μm	4 μm	5 μm	6 μm
p-stop Pos = 6 μm	Pre-irradiation	205 V	181 V	166 V	153 V	143 V
	Surface damage	103 V	121 V	128 V	132 V	118 V
	Surface + bulk damage	227 V	220 V	211 V	204 V	195 V
p-stop Pos = 8 μm	Pre-irradiation	248 V	224 V	208 V	194 V	182 V
	Surface damage	121 V	128 V	137 V	143 V	130 V
	Surface + bulk damage	256 V	244 V	234 V	226 V	216 V
p-stop Pos = 10 μm	Pre-irradiation	293 V	276 V	258 V	242 V	226 V
	Surface damage	127 V	137 V	146 V	148 V	133 V
	Surface + bulk damage	270 V	264 V	259 V	249 V	240 V
p-stop Pos = 12 μm	Pre-irradiation	299 V	292 V	282 V	270 V	257 V
	Surface damage	131 V	144 V	150 V	137 V	122 V
	Surface + bulk damage	276 V	271 V	267 V	261 V	254 V
p-spray	Pre-irradiation	134 V				
	Surface damage	264 V				
	Surface + bulk damage	248 V				

## Data Availability

The original contributions presented in this study are included in the article. Further inquiries can be directed to the corresponding author.
